# Method to Convert Stem Cells into Cancer Stem Cells

**DOI:** 10.3390/mps2030071

**Published:** 2019-08-16

**Authors:** Said M. Afify, Ling Chen, Ting Yan, Anna Sanchez Calle, Neha Nair, Chikae Murakami, Maram H. Zahra, Nobuhiro Okada, Yoshiaki Iwasaki, Akimasa Seno, Masahura Seno

**Affiliations:** 1 Department of Medical Bioengineering, Graduate School of Natural Science and Technology, Okayama University, Okayama 700-8530, Japan; 2 Division of Biochemistry, Faculty of Science, Menoufia University, Shebin El Koum-Menoufia 32511, Egypt; 3 Laboratory of Nano-Biotechnology, Graduate School of Interdisciplinary Science and Engineering in Health Systems, Okayama University, Okayama 700-8530, Japan; 4 Department of Gastroenterology and Hepatology, Graduate School of Medicine, Okayama University, Okayama 700-8558, Japan; 5 Okayama University Research Laboratory of Stem Cell Engineering in Detroit, IBio, Wayne State University, Detroit, MI 48202, USA

**Keywords:** cancer stem cells, induced pluripotent stem cells, embryonic stem cells (ESCs), conditioned medium (CM)

## Abstract

The cancer stem cell (CSC) hypothesis suggests that tumors are sustained exclusively by a small population of the cells with stem cell properties. CSCs have been identified in most tumors and are responsible for the initiation, recurrence, and resistance of different cancers. In vitro CSC models will be of great help in revisiting the mechanism of cancer development, as well as the tumor microenvironment and the heterogeneity of cancer and metastasis. Our group recently described the generation of CSCs from induced pluripotent stem cells (iPSCs), which were reprogrammed from normal cells, and/or embryonic stem cells (ESCs). This procedure will improve the understanding of the essential niche involved in cancer initiation. The composition of this cancer-inducing niche, if identified, will let us know how normal cells convert to malignant in the body and how, in turn, cancer prevention could be achieved. Further, once developed, CSCs demonstrate the ability to differentiate into endothelial cells, cancer-associated fibroblasts, and other phenotypes establishing the CSC niche. These will be good materials for developing novel cancer treatments. In this protocol, we describe how to handle mouse iPSCs/ESCs and how to choose the critical time for starting the conversion into CSCs. This CSC generation protocol is essential for understanding the role of CSC in cancer initiation and progress.

## 1. Introduction

Cancer remains a major cause of mortality all over the world. Statistics have predicted new cancer cases more than 20 million worldwide each year by 2030 [1]. Therefore, early diagnosis and treatment of cancer are currently the most critical issue for cancer prevention.

Understanding the mechanism of cancer initiation will provide us with critical clues in finding ways to treat cancer. However, the mechanisms underlying cancer initiation appear extremely complicated, with various phenotypes and numerous signaling pathways involved.

Many studies have defined the functional role of cancer stem cells (CSCs) in cancer initiation [2]. CSCs are considered to critically influence tumorgenicity [3] and metastasis [4] as well as provide resistance against therapeutic agents [5]. CSCs are postulated to be originate from normal stem cells [6]. This conversion may result from chronic inflammation, which continuously imbalances homeostasis, resulting in the generation of an irregular microenvironment surrounding progenitor cells or stem cells.

Induced pluripotent stem cells (iPSCs)/embryonic stem cells (ESCs) could acquire the characteristics of CSCs when cultured in the presence of conditioned medium (CM) prepared from various cancer cell lines without transducing foreign genes and/or mutagens [6,7,8,9,10,11,12,13,14,15]. The CSCs established from iPSCs through epigenetic regulations have been shown to have the capacity of self-renewal, differentiation, and malignant tumorigenicity. Based on our data, we summarize the detailed procedure and the critical conditions step-by-step.

## 2. Experimental Design

**Figure 1:**
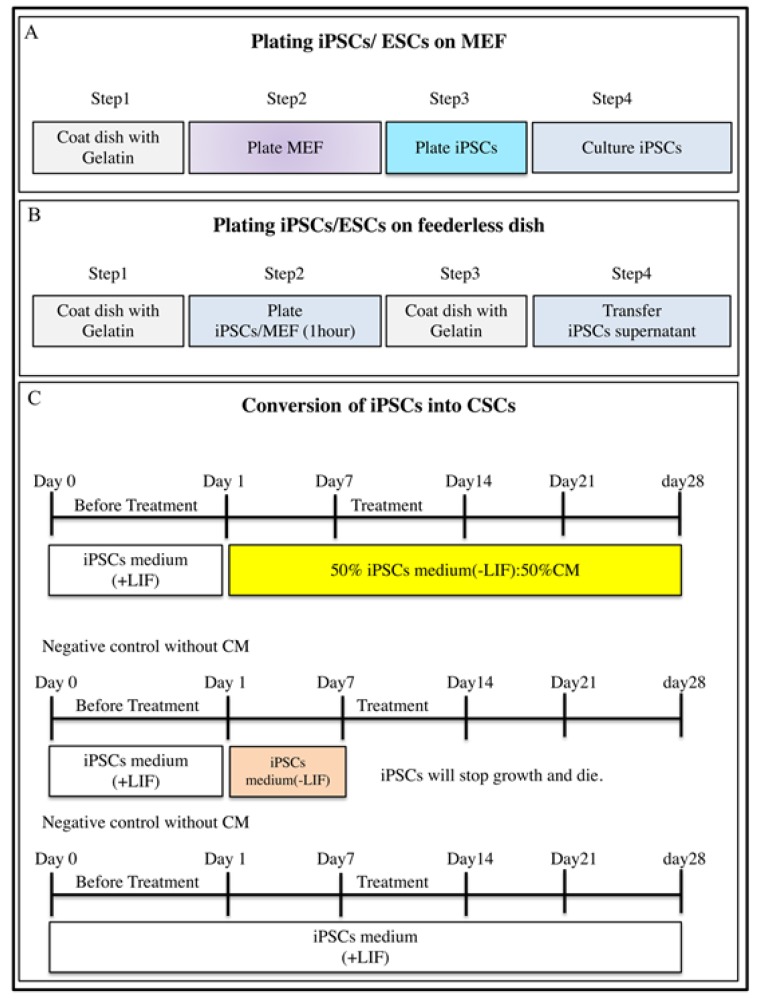
Representative scheme for conversion of stem cells into cancer stem cells. (**A**) Steps for plating induced pluripotent stem cells (iPSCs)/embryonic stem cells (ESCs) on mitomycin C-treated mouse embryonic fibroblast (MEF) feeder cells. (**B**) Steps for plating induced pluripotent stem cells (iPSCs)/embryonic stem cells (ESCs) on feederless gelatin-coated dishes. (**C**) Representative scheme for conversion of induced pluripotent stem cells (iPSCs)/embryonic stem cells (ESCs) into cancer stem cells (CSCs) in the presence of conditioned medium (CM) from cancer cell lines, and negative control without CM.

This protocol describes how to generate CSCs from iPSCs/ESCs in the presence of microenvironments conferred by different cancer cell lines. The protocol shows detailed experimental steps related to the conversion of stem cells into CSCs. First of all, CM was collected from a confluent culture of cancer cell lines. Then, iPSCs were converted into CSCs in the presence of CM, as summarized in Figure 1. Afterwards, the surviving cells were suspended in Hank’s balanced salt solution (HBSS) and injected into BALB/c nude mice. The surgical procedure was performed under sterile conditions. Almost one month later, malignant tumors developed in mice injected with the surviving cells, while teratoma developed in mice injected with mouse iPSCs/ESCs. Primary cultures from tumors were assessed for sphere formation to confirm characteristics of CSCs. The differentiation potential was confirmed by the appearance of a phenotype that was adhesive to the dishes. Ethanol (70%) was used for surgical instruments to maintain sterile conditions during the procedure. All animal experiments were reviewed and approved by the ethics committee for animal experiments of Okayama University under the ID OKU-2018078.

### 2.1. Materials

- Lewis lung carcinoma LL/2 (LLC1) (ATCC^®^ CRL-1642™);
- Breast cancer cell lines, T47D (ATCC ^®^ HTB-133);
- Liver cancer cell line PLC/PRF/5 (ATCC^®^ CRL-8024™);
- Pancreatic carcinoma cell lines PK-8 and KLM-1 (RIKEN cell Bank, Tsukuba, Japan);
- Mitomycin C treated mouse embryonic fibroblast (MEF) cells (REPROCELL Inc., Kanagawa, Japan);
- Mouse ESCs B6G-2 cells (RBRC-AES0003) Riken Cell Bank, Japan;
- Mouse iPSCs (iPS-MEF-Ng-20D-17, Lot No. 012, Riken Cell Bank, Tokyo, Japan), in which puromycin (puro) resistant gene and green fluorescent protein (GFP) gene were cloned under the control of Nanog promoter;
- Dulbecco’s modified Eagle’s medium–high glucose (Wako, Osaka, Japan (catalog number: 044-29765));
- L-Glutamine (Nacalai Tesque, Kyoto, Japan, ((catalog number: 16948-04));
- MEM Non-essential amino acid solution (100×) (Wako, Osaka, Japan, catalog number: 139-15651);
- 2-Mercaptoethanol (Sigma-Aldrich, St. Louis, MO, USA);
- Leukemia inhibitory factor (LIF, Merck Millipore, Burlington, MA, USA);
- Trypsin-EDTA (0.25%) (Nacalai Tesque, Kyoto, Japan, Cat. No: 327777-44);
- Fetal bovine serum (FBS, Gibco, Life Technologies, Massachusetts, USA,Cat. No: 10437-028);
- Penicillin/streptomycin mixed solution (100 U/mL) Nacalai Tesque, Kyoto, Japan, Cat. No-26253-84);
- 70% ethanol (Sigma-Aldrich; Cat. No.: 459836-2);
- Isoflurane (gas anesthesia system) DS Pharma Animal Health Co., Ltd. Osaka 541-0053, Japan);
- BALB/c-nu/nu, female, 4 weeks old, Charles River laboratories, kanagawa, Japan.

### 2.2. Equipment

- Eppendorf Centrifuge 5415R, Eppendorf AG, 22331 Hamburg, Germany;
- Sanyo MCO-19AIC(UV) CO_2_ Incubator, Marshall Scientific, Hampton, USA;
- Labculture^®^ Class II, Type A2 Biological Safety Cabinets (E-Series);
- Olympus IX81 microscope (Olympus, Tokyo, Japan);
- Laser scanning confocal microscope, FV-1000, Olympus, Tokyo, Japan;
- Tissue culture-treated plate, 60 mm dish; TPP Techno Plastic Products AG Schaffhausen, Switzerland, Cat. No 93060;
- Tissue culture-treated plate,100 mm dish; TPP Techno Plastic Products AG Schaffhausen, Switzerland, Cat. No 93100;
- Filter max 250 mL, TPP, Switzerland, Cat. No 99255;
- Falcon^®^ Conical Centrifuge Tubes (15 mL; BD Falcon, New York, USA Cat. No 352095);
- Falcon^®^ Conical Centrifuge Tubes (50 mL; BD Falcon, New York, USA Cat. No 352070);
- Liquid N_2_ storage tank.

## 3. Procedure

### 3.1. Conditioned Medium (CM) Preparation for Mouse iPSC/ESC Conversion

In our protocol, we used conditioned medium (CM) derived from cancer cell lines to convert stem cells to CSCs.

- Take of a vial of each cancer cell line from liquid nitrogen storage.
- Revive the cells into a 100 mm dish containing 5 mL Dulbecco’s modified Eagle’s medium (DMEM) supplemented with 10% FBS and 100 U/mL penicillin/streptomycin.
- Passage cells again after a couple of days, when the cells are 70% confluent.
- Prior to collecting conditioned medium, change the medium to 5% FBS at 80% confluency.

**Critical:** Cells should be at least 80% confluent.

- After incubation for 48 h, collect the conditioned medium (CM) from Huh7 cells from confluent dishes.

**Critical:** Precaution should be taken to avoid overgrowth.

- Centrifuge at 300*g* for 10 min.
- Separate the supernatant in a new tube and filters using 0.22 mm filter.
- Remove 2 mL CM then add this to a 3.5 cm dish overnight to confirm there are no surviving cancer cells in CM.
- Store CM at −20 °C for later experiments.

### 3.2. Mouse iPSC/ESC Handling

#### 3.2.1. Plating Mitomycin C-Treated Mouse Embryonic Fibroblasts (MEFs)

Our group revived iPSCs/ESCs on mitomycin C-treated mouse embryonic fibroblast (MEF) feeder cells for the maintenance the undifferentiated state of iPSCs/ESCs.

- Add 2 mL sterile 0.1% gelatin to cover the bottom of 6 dishes.
- Incubate the gelatin-coated dishes for at least 30 min at 37 °C.
- Remove the MEF vial from the liquid nitrogen and thaw quickly in a 37 °C water bath.
- Remove the vial from the water bath after the vial is half-thawed.
- Sterilize the tube by spraying with 70% ethanol.
- Transfer the cells with 5 mL of DMEM medium containing 10% FBS to a 15 mL conical tube.
- Pellet the cells by centrifugation at 100*g* for 5 min.
- Discard the supernatant.
- Resuspend the pellet with 3 mL fresh MEF medium.
- Aspirate excess gelatin solution from the incubated dishes.
- Add 4 mL DMEM contains 10% FBS medium (37 °C) to the dish.
- Plate the cells on gelatin-coated plates at seed density of 6 × 104 cells/6 cm^2^.
- Incubate at 37 °C with 5% CO_2_, until the cells reach 80%–90% confluency.
- Keep monitoring the cells every day.
- Change the medium twice a week.

#### 3.2.2. Plating Mouse iPSCs/ESCs

Our group grew iPSCs/ESCs on MEF feeder cells using iPSCs complete medium in the presence of leukemia inhibitory factor (LIF). It is important that iPSCs/ESCs be subcultured every 4 days at a low density to maintain their growth in the exponential phase. Under carefully monitored iPSCs/ESCs culture conditions, iPSCs/ESCs maintain pluripotency and self-renewing capacity. After transferring iPSCs/ESCs to a gelatin-coated dish, cells should be monitored until forming separate colonies without differentiation. The time at which colonies become 70% confluent is considered to be the critical time for starting conversion.

- Prepare iPSCs complete media as described above.
- Pre-warm mouse iPSC/ESC medium in a 37 °C water bath for 30 min.

**Critical:** Do not keep the media in the water bath for more than 1 h at 37 °C as continued exposure to 37 °C will reduce the effectiveness of the growth factors.

- Replace the MEF medium with 4 mL of iPSCs complete medium (+LIF).
- Remove frozen mouse iPSCs/ESCs from liquid nitrogen storage.
- Thaw the cells by gently swirling in a 37 °C water bath.
- Sterilize the vial with 70% ethanol.
- Add 5 mL iPSCs medium to a 15 mL conical tube.
- Use a 1 mL pipette to gently transfer the cell suspension to a 15 mL conical tube.
- Shake the conical tube gently to mix the cells.
- Centrifuge cells at 100*g* for 5 min at room temperature.
- Aspirate the supernatant and discard.
- Add 5 mL of iPSCs medium.
- Gently resuspend the pellet by pipetting up and down 2 or 3 times with a 1 mL tip.
- Seed 0.1 × 10^6^ of cells onto 6 cm MEF dishes.

**Critical:** Avoid seeding mouse iPSCs/ESCs at high density because they tend to aggregate and give rise to cells with mixed morphologies.

**Figure 2:**
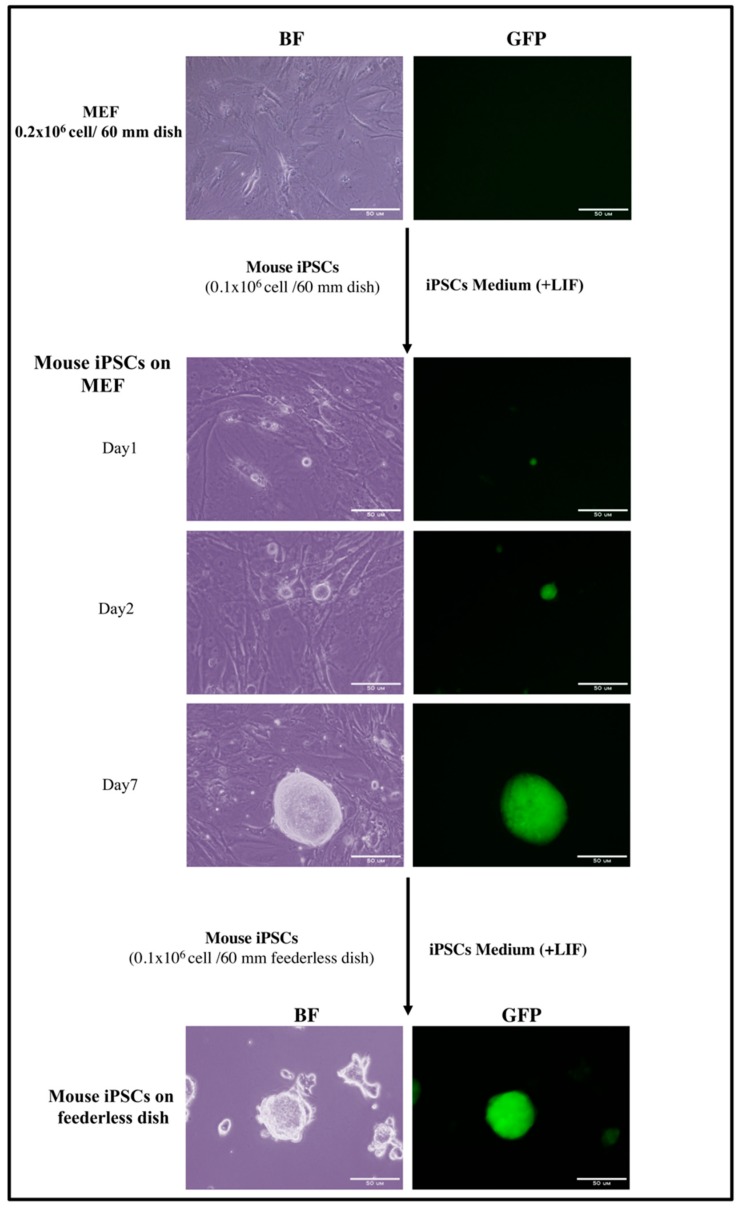
Representative images of mouse induced pluripotent stem cell (iPSC)/embryonic stem cell (ESC) viability maintenance in the presence of leukemia inhibitory factor (LIF). iPSCs/ESCs seeded om MEF feeder cells for at least one week until forming colonies without differentiation. These colonies were transferred to feederless gelatin-coated dish prior to start conversion. Stemness tracking during maintenance by the presence of GFP protein. Scale bar: 50 μm.

- Change the medium the next day to remove the dead cells and daily thereafter until the cells have been cultured for 7 days or the colonies reach 80% confluency (Figure 2).

### 3.3. Conversion of Mouse iPSCs/ESCs into CSCs

To initiate the conversion of iPSCs/ESCs into CSCs, we used CM derived from a cancer cell line. We suppose that chronic inflammatory conditions trigger stem cells to develop into CSCs [6]. During this process, cytokines and other soluble mediators in the tumor microenvironment (CM) bound to stem cell surface receptors stimulate the intracellular signaling cascade in order to direct stem cell fates into the CSCs phenotype. We recommend using conversion medium containing CMs and iPSCs complete medium at a 1:1 ratio in order to convert stem cells into CSCs.

- Cover the bottom of 6 dishes with 2 mL sterile 0.1% gelatin for each dish.
- Incubate the gelatin-coated dishes for at least 30 min at 37 °C.
- Trypsinize mouse iPSC/ESCs MEF dishes and collect in 15 mL tube.
- Centrifuge cells at 100*g* for 5 min at room temperature.
- Aspirate the supernatant.
- Resuspend the cell pellet in 3 mL iPSCs complete medium.
- Aspirate excess gelatin from 3 dishes.
- Add 4 mL iPSCs complete medium in each dish.
- Add 1 mL from mouse iPSCs/ESCs in each dish.
- Incubate for 1 h at 37 °C with 5% CO_2_.

**Critical:** Keep the cells for only 1 h to let the differentiated cell settle down. After 1 h, only the undifferentiated mouse iPSCs/ESCs are present in the medium.

**Figure 3:**
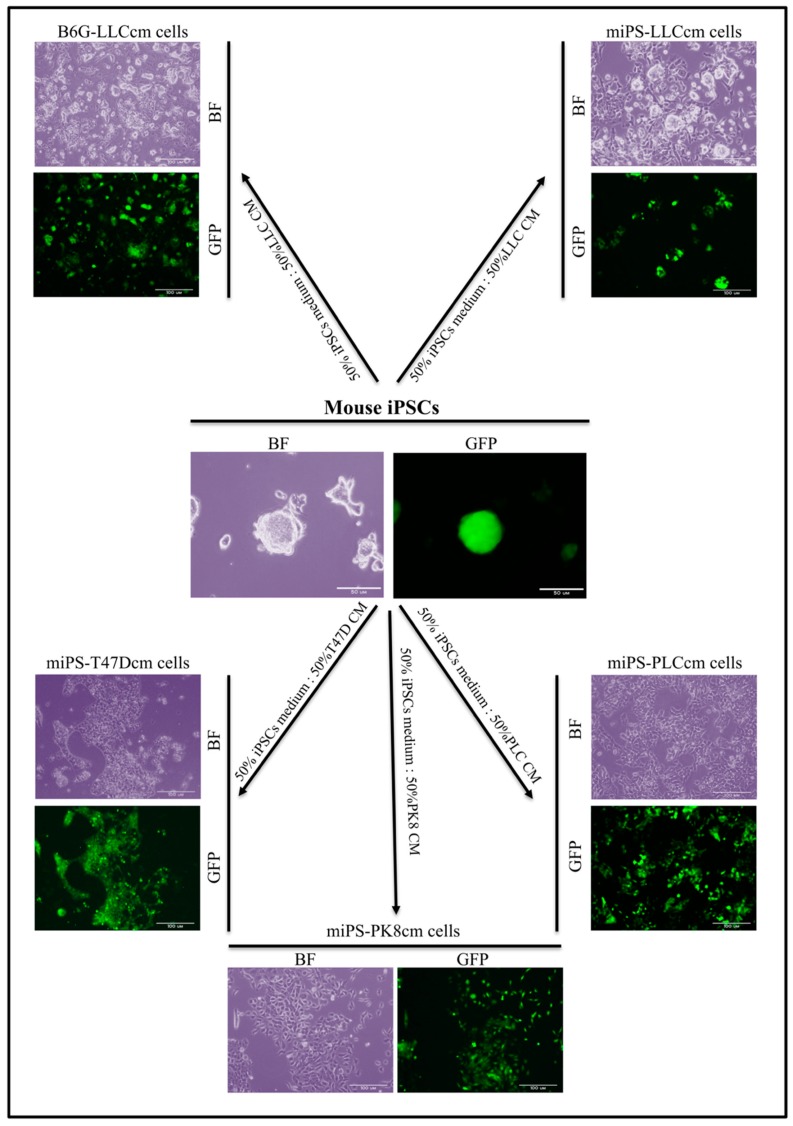
Summarized scheme for mouse induced pluripotent stem cells (iPSC)/embryonic stem cell (ESC) conversion in the presence of conditioned medium (CM). CM from different cancer cell lines (Lewis lung carcinoma LL/2 (LLC1), breast cancer cell line (T47D), liver cancer cell line (PLC/PRF/5), pancreatic carcinoma cell line (PK-8)) induce the conversion of iPSCs/ESCs into cancer stem cells after 4 weeks of treatment. Stemness tracking during conversion by the presence of GFP protein. Scale bar: 100 μm.

- Transfer the supernatant containing the mouse iPSCs/ESCs to the 3 other gelatin-coated dishes.
- For the initiation of CSCs, at 70% confluency, change the medium to a conversion medium (iPSCs medium combined with the 50% CM).
- Change the medium every 48 h.
- Subculture the cells every 4 days.
- Keep conversion for four weeks.
- Monitor the conversion using GFP protein if controlled under a Nanog promoter and photograph the cells every week using Olympus IX81 microscope equipped with fluorescence (Figure 3).

### 3.4. CSCs Transplantation

- Autoclave all surgical materials, suture thread, suture needle, and cotton.
- Set all surgical materials in the laminar hood.
- Turn UV lamp for 20 min on all materials.
- Turn off UV lamp and turn on light.
- Divide the mice into six groups, three mice for each type of converted cells and mouse iPSCs/ESCs as the control.
- Anesthetize the animals with 2% isoflurane in an anesthesia chamber.
- Disinfect with 70% ethanol and then iodine.
- Cut an area of 2–3 cm^2^.
- Draw up 50 μL of phosphate-buffered saline with 0.5 × 10^6^ of the cells into a 26 G × 5/8″ syringe.
  - Inject miPS-LLCcm cells subcutaneous [7].
  - Inject miPS-PK8cm cells in the pancreas [9].
  - Inject miPS-T47Dcm cells in MFP [10].
  - Inject miPS-PLCcm cells into the liver.
  - Inject B6G-LLCcm cells subcutaneously [15].
- Inject the cells slowly into the organ or subcutaneous until it is completely injected.

**Critical:** Cells should be infused very slowly.

- Wait for 60 s.

**Critical:** Do not remove the needle immediately after infusion to avoid the backflow of cells as well as bleeding. After 1 min, clotting will occur at the site of puncture.

- Remove the needle.
- Close the abdominal peritoneum and then skin with the degradable sterile suture separately.
- Keep the mice in a pre-warmed cage directly after surgery for recovery until the mouse shows regular breathing patterns.
- Allow free access to water and food.

### 3.5. Malignant Tumor Detection and Primary Culture

- After 30 days of transplantation, anesthetize the mice with 2% isoflurane in an anesthesia chamber.
- Sacrifice the mice.
- Remove the tumors using scissors.
- Excise the tumors then fix in 10% neutral formalin buffer solution for hematoxylin and eosin Y staining and immunohistochemical analysis.
- Cut the rest of malignant tumor derived from different converted cells into small pieces.
- Take small pieces of each tumor (approximately 1 mm^3^ size) and wash in the HBSS three times.
- Transfer these pieces into a 15 mL tube with 4 mL of dissociation buffer for 6 h.
- Terminate the digestion with 5 mL of DMEM containing 10% FBS.
- Transfer the cellular suspension into the new tubes.
- Centrifuge at 100*g* for 5 min.
- Resuspend the cells pellet in 5 mL medium.
- Centrifuge at 100*g* for 5 min.
- Place the cell pellet into an appropriate volume of iPSCs complete medium without LIF.
- Seed the cells into a dish at a density of 1 × 10^5^ cells/cm^2^.
- After one day, remove the medium and change new one supplemented by puromycin to remove any cell from the host.
- Observe the expression of GFP and cell morphology, then photograph using Olympus IX81 microscope equipped with a light fluorescence device.
- Subject the primary cultured cells to RT-qPCR analysis in order to check the gene expression in comparison with mouse iPSCs.
- Check self-renewal potential of new derived primary cultured cells by sphere formation assay.

### 3.6. Sphere Formation Assay

- After maintaining the primary culture from tumor and treating with puromycin, trypsinize the cells and count to perform the sphere formation assay.
- Pre-warm mouse sphere medium in a 37 °C water bath for 30 min.
- Centrifuge for 10 min at 100*g* at room temperature (RT).
- Discard the supernatant.
- Dissociate the pellet by pipetting in a serum-free medium.
- Count the cells and take the required number of live cells.
- Seed the cells in sphere medium, prepare at least 4–6 replicates for each primary culture.

**Critical:** The seeded cells should appear as singlets.

- Place the plate in an incubator set at 37 °C with 5% CO_2_.
- Wait for 7 days then count the sphere under an optical microscope.

## 4. Expected Result

**Figure 4:**
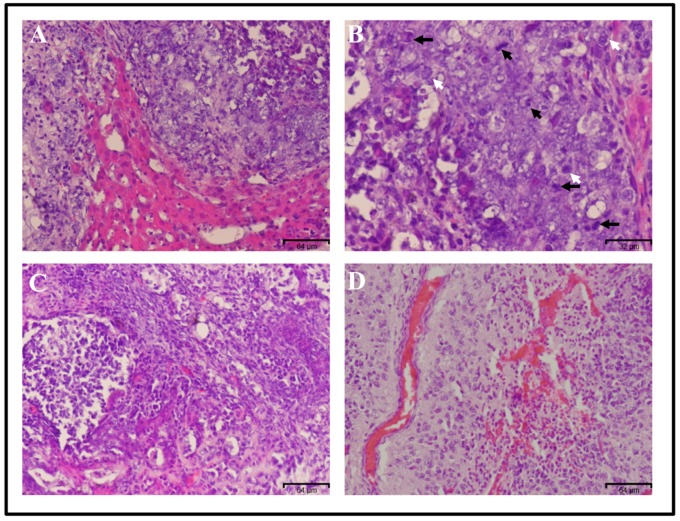
Histological analysis of the tumor derived from transplantation of miPS-PLCcm cells. Hematoxylin and eosin staining showed tumor invasion inside the normal liver (**A**). Original magnification 20×. This tumor tissue showed malignant phenotype criteria such as mitotic figures (black arrows), nuclear atypia (white arrows) (**B**) Original magnification 40×, high nuclear to cytoplasmic ratio (**C**) and angiogenesis (**D**). Original magnification 20×.

The protocol described here outlines a technique utilized for the induction of CSC using CM derived from different cancer cell lines. The converted cells are highly tumorigenic with malignancy while mouse iPSCs/ESCs form benign teratoma. All the tumors derived from converted cells showed the malignancy, for example, the tumor formed by miPS-PLCcm cells exhibited malignancies such as mitotic figures, nuclear atypia, high nuclear to cytoplasmic ratio and angiogenesis (Figure 4).

**Figure 5:**
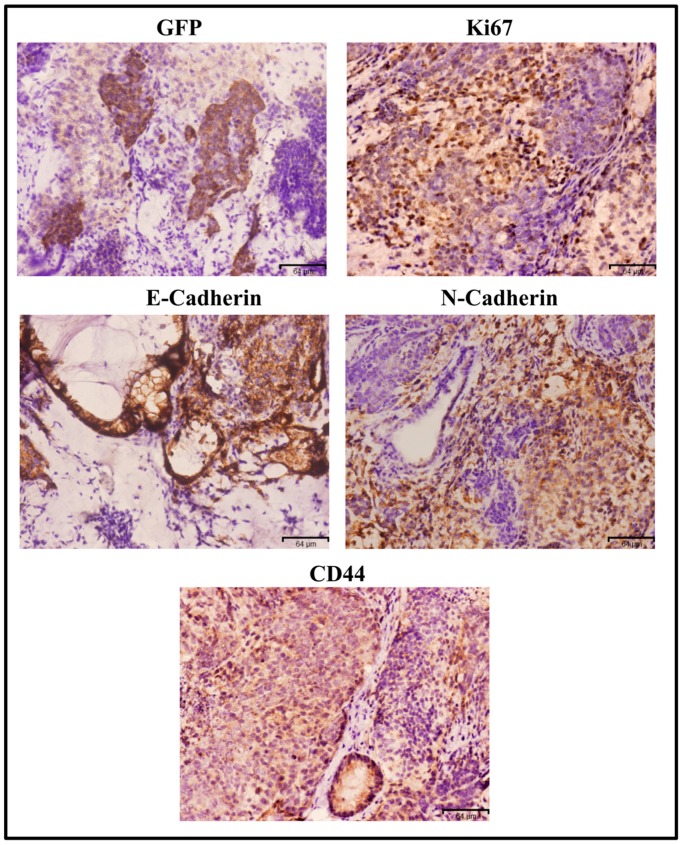
Immuno-histochemistry (IHC) of miPS-PLCcm cells derived tumor in the liver. The tissue section was stained with antibodies against CD44, Ki67, GFP, N-cadherin, and E-cadherin, original magnification 20×.

The malignant tumor derived from miPS-PLCcm cells was assessed for several markers by immunohistochemistry (Figure 5). GFP staining indicated undifferentiated population derived from miPS-PLCcm cells. Strong immunoreactivity to Ki67 indicated high proliferation rate in the tumor tissue. Immunoreactivity to CD44, E-cadherin and N-cadherin implied the heterogeneous phenotypes in malignant tumor derived from miPS-PLCcm cells.

**Figure 6:**
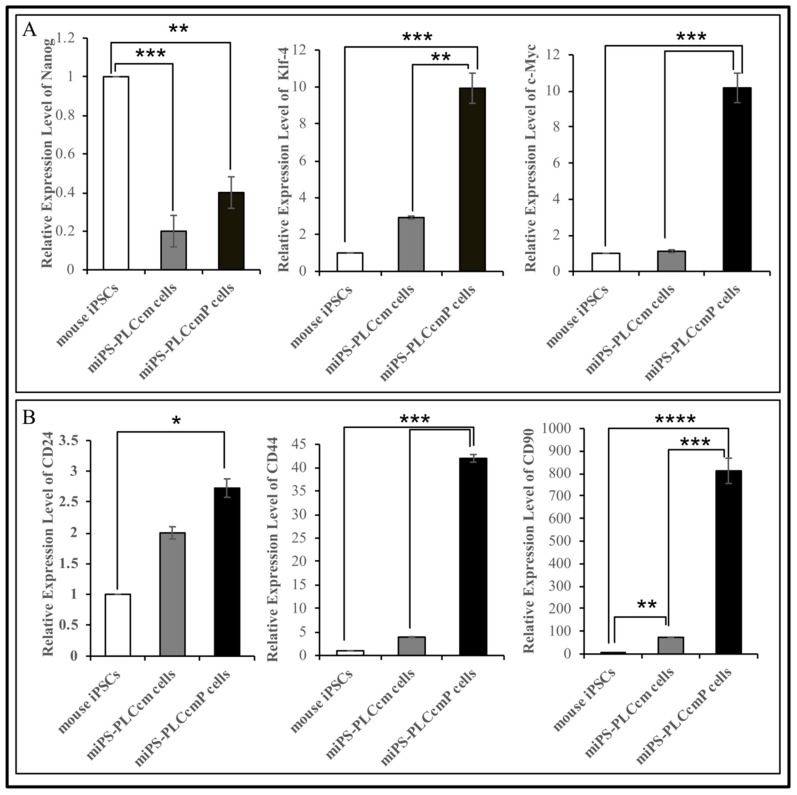
Characterization of cancer stem cells derived from iPSCs in the presence of conditioned medium derived from PLC cells and the primary culture derived from developed the malignant tumor. (**A**) RT-qPCR analysis of stemness markers in miPS-PLCcm and miPS-PLCcm P cells and in comparison, with miPSCs.; (**B**) RT-qPCR analysis of CSC markers in miPS-PLCcm and miPS-PLCcm P cells in compassion with mouse iPSCs.

The converted cells and the primary from all derived tumors sustained the expression of stemness and CSCs markers. For example, miPS-PLCcm cells and miPS-PLCcmPcells sustained the expression of stemness markers such as Nanog, Klf-4, and c-Myc as well as acquired CSC markers such as CD24, CD44, and CD90 (Figure 6).

**Figure 7:**
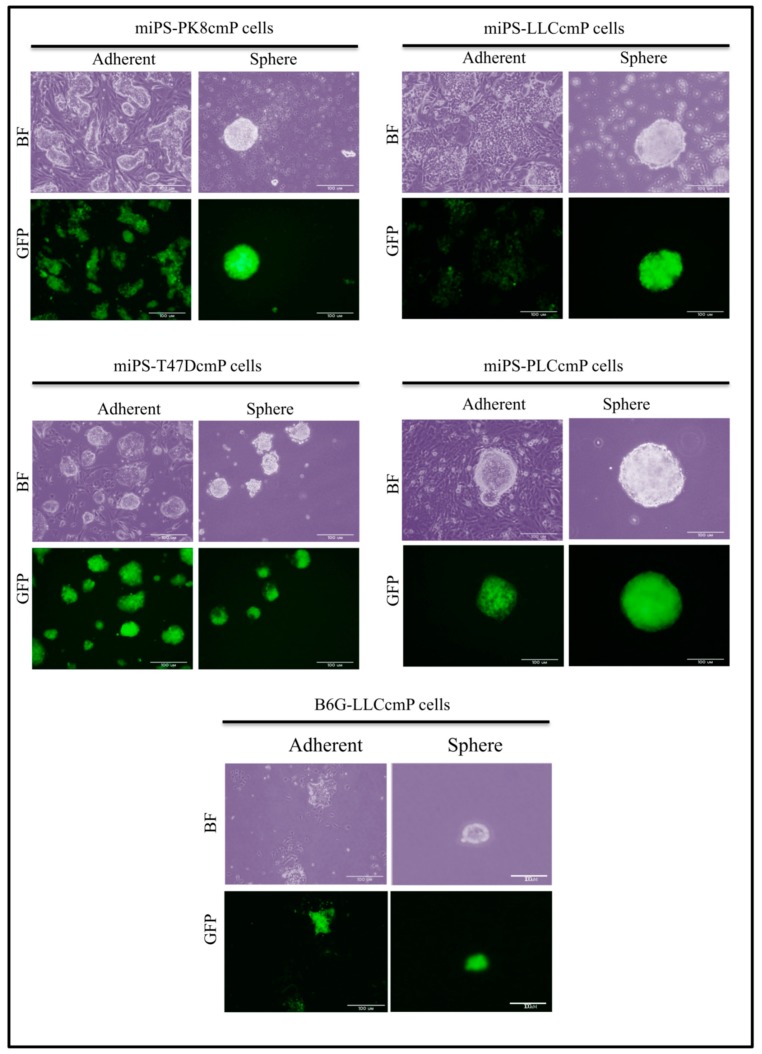
Representative images of differentiation and self-renewal potential of primary culture cells derived from malignant tumor.

These primary cells in an adhesive culture will exhibit two subpopulations: one is a colony, expressing GFP if directed by a Nanog promoter, surrounded by the other, which is epithelial-like cells with suppressed GFP expression by an inactive Nanog promoter. Simultaneously, these cells will also exhibit self-renewal potential in a sphere formation assay (Figure 7).

Primary cultures cells from different tumors derived from converted cells: miPS-LLCcmP cells, miPS-PK8cm P cells, miPS-T47DcmP cells miPS-PLCcmP cells, and B6G-LLCcmP cells exhibited two subpopulations: one is a colony expressing GFP and epithelial-like cells surrounding it in adhesive condition and with self-renewal potential in a non-adherent culture condition. Scale bars represent 100 μm.

## 5. Conclusions

Our group successfully established a protocol to generate CSCs with self-renewal, differentiation, and tumorigenic potential. This model of CSCs forms malignant tumors within only one month after injection. These CSCs—converted from iPSCs/ESCs in the microenvironment produced by cancers—will be a novel resource for evaluate cancer development and treatment in new ways.

## 6. Reagents Setup

### 6.1. Phosphate-Buffered Saline (PBS)

Prepare 400 mL of distilled water in a suitable container, then add 4 g of NaCl, 100 mg of KCl, 0.72 g of Na_2_HPO_4_, and 120 mg of KH_2_PO_4_ to the solution. Adjust solution to the desired pH (typically pH ≈ 7.5) then finally add distilled water until the volume is 0.5 L.

### 6.2. Gelatin

Prepare a 0.1% gelatin solution by dissolving gelatin in milliQ water and then sterilize by autoclaving at 121 °C for 30 min. The solution should be stored at room temperature 2–8 °C.

### 6.3. MEF Medium

Prepare high-glucose Dulbecco’s modified Eagle’s medium (DMEM) supplemented with 10% FBS, 2 mM glutamine, 1 × 10^−4^ M nonessential amino acids, and 50 U/mL penicillin/streptomycin.

6.4. iPSCs Complete Medium

Prepare high-glucose Dulbecco’s modified Eagle’s medium (DMEM) supplemented with 15% fetal bovine serum (FBS), 0.1 mM non-essential amino acid (NEAA, Life Technologies), 2 mM l-glutamine, 0.1 mM 2-mercaptoethanol, 1000 U/mL leukemia inhibitory factor (LIF, Milli- pore), 50 U/mL penicillin, and 50 U/mL streptomycin.

### 6.5. Conversion Medium

Mix an equal volume of CM and iPSC complete medium. Change this medium every 2 days.

### 6.6. Dissociation Buffer

Prepare PBS supplemented with 0.25% trypsin, 0.1% collagenase, 20% KnockOut™ Serum Replacement (Gibco, NY), and 1 mM of CaCl2 and keep at −20 °C.

### 6.7. Sphere Medium

Prepare serum free medium (DMEM 97.5%) supplemented with NEAA 1%, l-glutamine 1%, 100× penicillin/streptomycin 0.5%, 0.1 mM 2-mercaptoethanol, and insulin–transferrin–selenium-100X 1/100 v/v.
